# Synthesis, Characterization, Antibacterial and Anti-Inflammatory Activities of Enoxacin Metal Complexes

**DOI:** 10.1155/2009/914105

**Published:** 2009-08-02

**Authors:** Saeed Arayne, Najma Sultana, Urooj Haroon, M. Ahmed Mesaik

**Affiliations:** ^1^Department of Chemistry, University of Karachi, Karachi-75270, Pakistan; ^2^Department of Pharmaceutical Chemistry, Faculty of Pharmacy, University of Karachi, Karachi-75270, Pakistan; ^3^PCMD, International Centre of Chemical Sciences, University of Karachi, Karachi-75270, Pakistan

## Abstract

The present work comprises the synthesis of enoxacin (Heno) complexes with various transition metals. Two types of complexes [M(eno)_2_(H_2_O)_2_]3H_2_O(M = Cu^II^, Ni^II^ or Mn^II^) and [M(eno)(H_2_O)_2_]Cl · 4H_2_O (M = Fe^III^) were obtained. The complexes were characterized by different physicochemical, spectroscopic, and elemental analysis. Results suggest that enoxacin interacts with the metals as a monoanionic bidentate ligand. These complexes were also tested for their antibacterial activity against eleven (11) different microorganisms, and the results were compared with the parent drug. Moreover all the metal complexes were also tested for their ability to scavenge reactive oxygen species where by Mn^II^ and Cu^II^ complexes exhibited potential to mediate anti-inflammatory response.

## 1. Introduction

**Figure 1:**
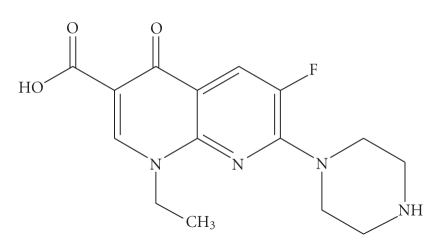
Enoxacin.

Enoxacin,1-ethyl-6-fluoro-1,4-dihydro-4-oxo-7-(1-pipera-zinyl)-1,8-naphthyridine-3-carboxylic acid is a potent inhibitor of the bacterial enzyme DNA gyrase, exhibiting high antibacterial activity against a broad spectrum of *Gram-negative * and moderate activity against *Gram-positive * bacteria [1–3] (Figure 1).

The most important structural features necessary for meaningful antibacterial activity of enoxacin include a carboxylic acid attached to the 3-position of the quinolone nucleus and an alkyl group in the 1-position. In addition to this, fluorine attached to the 6-position and a nitrogen heterocycle attached to the 7-position is also required for their activity. This heterocycle in enoxacin is a piperazine derivative [4]. Isosteric replacements of nitrogen for carbon atom at postion 8 (1,8-napthyridines) are consistent and retained the antimicrobial activity [5].

Quinolones are complexing agents for a variety of metal ions including alkaline earth and transition metal ions. Although reports indicate that the coordination of quinolones to metal ions such as Cu^II^, Mg^II^, and Ca^II^ appear to be important for the activity of the quinolone antibiotics [6], it has a detrimental effect on their absorption [7]. Early studies by Nakano demonstrated the ability of the quinolone naldixic acid to complex a variety of metal ions [8]. The crystal structures of quinolone complexes [9, 10] indicate that quinolone antibiotics can participate in the formation of complexes in a number of ways. Complexes isolated from acidic media usually contain singly and/or doubly protonated quinolones that are incapable of bonding to the metal ion and in these cases only electrostatic interaction was observed between the drug and the metal ions [11, 12]. In other cases [13, 14], it was found that neutral quinolones in the zwitterionic state are capable of forming simple complexes. In these complexes the quinolone acts as a bidentate ligand through the ring carbonyl group at position 4 and through one of the oxygen atoms of the carboxylato group at position 3. Quinolones can also act as bridging ligands and thus capable of forming polynuclear complexes.

 Literature review reveals that there are few studies on the interaction of metal ions with enoxacin [15]. Accordingly, an attempt is made to study the interaction of enoxacin with transition metals of biological interest and to investigate the coordination chemistry of such interactions.

In the present work, we describe the synthesis and characterization of metal complexes of enoxacin. Moreover the antibacterial and anti-inflammatory activity of enoxacin metal complexes is also evaluated and compared with the parent. Results suggest that metal interaction in some cases significantly altered the activity of enoxacin.

## 2. Experimental

### 2.1. Physical Measurements

Prior to the synthesis of the metal complexes, the metal:drug ratios were determined by conductometric titration and continuous variation method (jobs plot). Conductometry was carried on Vernier LabPro. Data acquisition and analysis was carried out by using Logger pro 3.2 software. For jobs plot the UV-Vis spectra of the drug and metal solutions (in different combination ratios) were recorded on UV-Vis spectrophotometer (Shimadzu 1601 coupled with a P IV–PC and loaded with UVPC version 3.9, software). Thin layer chromatography (TLC) was performed on HSF-254 TLC plate and the samples were visualized under UV lamp. Melting point of the metal complexes was recorded on a Gallenkamp apparatus. The characterization of enoxacin metal complexes was carried out by Fourier Transform Infrared Spectrophotometer (Shimadzu Prestige-21 200 VCE), coupled to a P IV-PC and loaded with IR Resolution software. The disks were placed in the holder directly in the IR laser beam. Spectra were recorded at a resolution of 2 cm^−1^, and 50 scans were accumulated. NMR spectra were recorded on Bruker AMX 500 MHz spectrometer in CD_3_OD using TMS as an internal standard. Column chromatography was performed on Merck silica gel 60 (particle size 0.06–0.02). 

### 2.2. Synthesis of Metal Complexes

Enoxacin base was a kind gift from Zafa Pharmaceutical Laboratories Ltd., Karachi, Pakistan. The metal salts used were of analytical grade. All the glassware was washed with chromic acid followed by a thorough washing with deionized water which was freshly prepared in the laboratory.

Synthesis of metal complexes of enoxacin was carried out according to their calculated mole ratios. For this purpose enoxacin along with ferric chloride hexahydrate, manganese chloride monohydrate, nickel chloride hexahydrate, and copper chloride di hydrate were purified by recrystallization. Solvent used for the synthesis of these complexes was double distilled methanol.

The Fe^III^ complex was synthesized by dissolving 1 mmole (0.27 gm) of the metal salt and 1 mmole (0.32 gm) of the drug separately in equal volume of hot methanol (20 mL) and then mixing the two solutions with constant stirring in a round bottomed flask. The flask was fitted to water condenser, and the solution was refluxed for about an hour. The completion of the reaction was monitored by TLC. Solvent system used was water, ethyl acetate, and acetic acid in the ratio 1 : 2 : 1. In a similar way other metal complexes were synthesized taking 1 mmole (0.144 gm) of Mn^II^ salt, 1 mmole (0.2374 gm) of Ni^II^ salt, 1 mmole (0.161 gm) of Cu^II^ salt, and 2 mmole (0.64 gm) of the drug. The volume of the reaction mixture was then reduced by rotary evaporation. The precipitated complexes were filtrated off, washed with water and methanol and vacuum dried. The physical characteristics of enoxacin metal complexes are given in (Table 1).

### 2.3. Antibacterial Studies

Disk Diffusion technique developed by Bauer et al. [16] was adopted to determine the antibacterial activity of enoxacin and all the metal complexes against 11 different clinical isolates of *Gram-positive * and *Gram-negative * organisms, that is, *Klebsiella Pneumoniae, Proteus mirabilis, Staphylococcus aureus, Cornybacterium hoffmini, Shigella flexneri, Escherichia coli, Pseudomonas areuginosa, Bacillus species, Citrobacter species, Streptococcus pneumonia, * and *Salmonella typhi.*

The antibacterial activity of the compounds (ligand, metal salt, and complexes) was determined at a concentration range of 10 mg/disc, 15 mg/disc, and 20 mg/disc. All the solutions were prepared in hot methanol. The discs were applied on the surface of the agar plate over which a culture of microorganism was already streaked. After 24 hours of incubation the clear zone of inhibition around the disc was determined; this was related to the activity of the compound against the test strain. Three replicas were made for each treatment to minimize error.

### 2.4. Phagocyte Chemiluminescence

Luminol-enhanced chemiluminescence assay was performed using Helfand et al. protocol [17]. Briefly whole blood diluted in modified Hank's solution (MHS) was incubated with different concentrations of the metal complexes (50, 25, and 5 *μ*g/mL) for 30 minutes Zymosan (Sigma Chemical Co., USA) 100 *μ*L (20 mg/mL), followed by 100 *μ*L (7 × 10^5^ M) luminal (Sigma Chemical Co., USA) added to make a final volume of 0.25 mL.

MHS alone was run as a control. Peak chemiluminescence of the drug and the complexes was recorded with a luminometer (Labsystem Luminoskan RS, Finland). The luminometer was set with repeated scan mode, 50 scans with 30s intervals and one second point measuring time. The experiments were performed three times to minimize errors.

## 3. Result and Discussion

**Figure 2:**
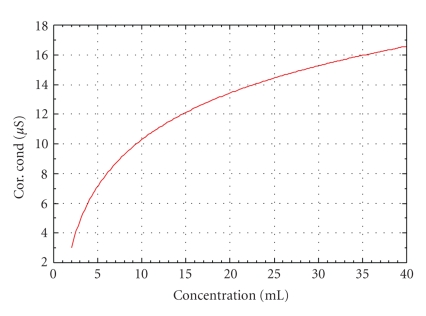
Representative conductance graph of Enoxacin and its Mn^II^ complex.

Enoxacin has a favorable solubility in acidic or basic solvents and in hot water. However the metal complexes of enoxacin were insoluble in hot water. Solubility was found in hot methanol, ethanol, and chloroform. The complexes were found to be stable at room temperature for two days [18]. The stability was checked by taking melting points of the complexes at an interval of 24 hours and 48 hours. All the samples were stored in room temperature (25°C). No appreciable changes in the melting points were observed, and the estimated error was ±1°C. Melting points of the metal complexes when compared with the reference drug (Table 1) differ considerably from enoxacin. Conductometric titration (Figure 2) and jobs plot reveal that the metal: drug complexes are in the ratio 1 : 1 (metal = Fe^III^) and 1 : 2 (metal = Cu^II^, Ni^II^ or Mn^II^). Argentometric titration of Iron(III) complex using Potassium dichromate as an indicator shows one molecule of chloride ion present outside the coordination sphere of the complex. The structure of the complexes established form the elemental analyses agree well with their proposed formulae (Table 2).

In the IR spectra of enoxacin, two strong absorption peaks at 1690 and 1640 cm^−1 ^ are observed due to carboxylic and ring ketonic (C=O) groups, respectively.

On comparing the IR spectrum of enoxacin with its metal complexes it is found that the band due to carboxylic group at (1690 cm^−1^) nearly diminishes in the spectra of the complexes indicating the coordination of this moiety to the metal ions [19]. Further the absorption of the ring ketone appears at a lower frequency near (1600 cm^−1^) in the spectra of the complexes which also suggests the binding of enoxacin to the metal ions through the ring carbonyl oxygen atom [20]. A broad diffuse band of medium intensity in the region (3100–3500 cm^−1^) is assigned to the OH stretching vibration of the water molecules [21].

Important ^1^H NMR signals of enoxacin are observed at chemical shifts of 1.40 (t, 3H, J = 7.0 Hz, –CH_3_ methyl), 2.0 (s, 1H, amine), 2.62 (s, 4H, piperazine), 3.85 (s, 4H, piperazine), 4.48 (q, 2H, J = 7.0 Hz, –CH_2_– ethyl), 8.10, 8.95 (s, 2H, naphthyridine), and 11.0 (s, 1H, COOH).

On comparing main peaks of enoxacin with its complexes, it is observed that all the signals of the free ligand are present in the ^1^HNMR spectra of the complexes. The signals for the aliphatic and piperazine protons are practically unchanged since they lie far from the binding site of the ligand [22]. However, resonance of naphthyridine proton from 8.10–8.95 appeared down field near 8.4–9.5 ppm in the spectra of all the complexes. Other studies also report that the aromatic protons exhibit a characteristic 0.3–0.5 ppm downfield shift upon binding of the quinolones to the divalent metal ion [23]. The resonance of the carboxylic proton (COOH) is not detected in the spectra of the complexes which further suggest the coordination of enoxacin through its carboxylate oxygen atoms [24]. The OH proton peak appears near 3.5 ppm, adjacent to the piperazine protons, due to the presence of lattice water.

**Figure 3:**
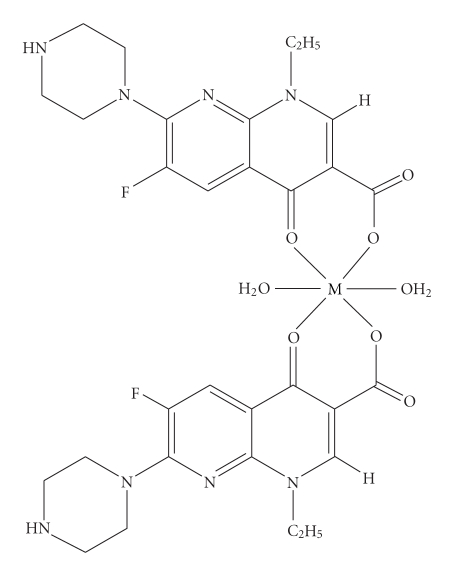
Tentative structure of Cu^II^, Mn^II^, and Ni^II^ complexes of enoxacin.

**Figure 4:**
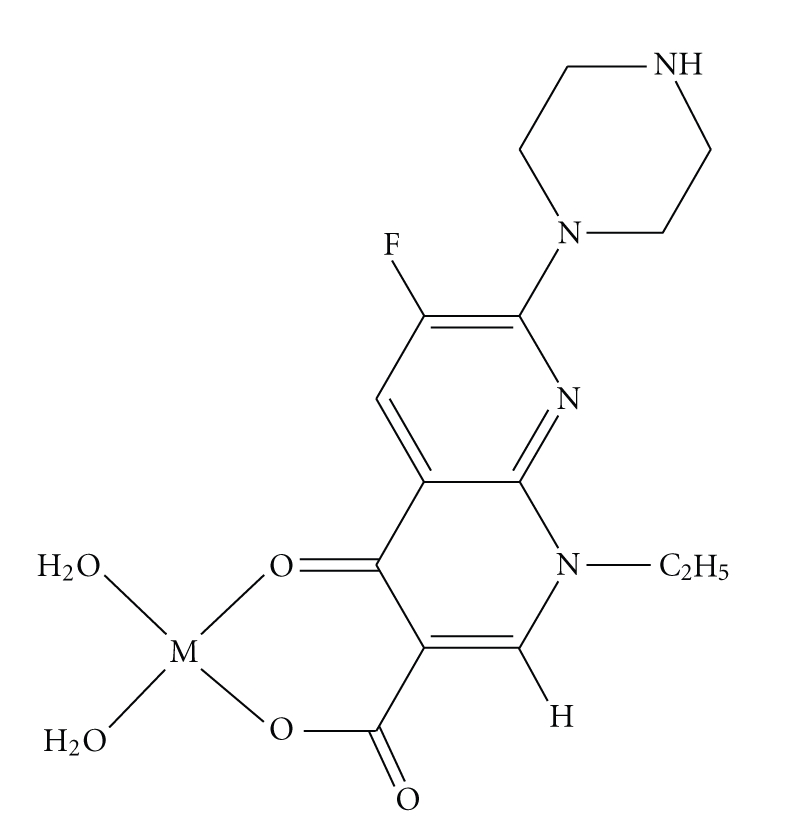
Tentative structure of Fe^III^ complex of enoxacin.

Our studies suggest that enoxacin acts as a monoanionic bidentate ligand and interacts with the metal centre through the 3-carboxylate and 4-oxygen atom. From the results obtained, it is proposed that the Manganese(II), Nickel(II), and Copper(II) complexes are probably six coordinate with two molecules of enoxacin chelating the central metal atom from four sides and two molecules of water at the vertices of an octahedron (Figure 3). Alternatively, the enoxacin complex of Iron(III) is four coordinate with probably one molecule of the drug and two molecules of water along the edge of a tetrahedron (Figure 4). Despite the crystalline nature of the products, we did not manage to obtain crystals suitable for determination of structures with X-ray crystallography [21, 22]. 

### 3.1. Antibacterial Studies

The zone of inhibition around the antibiotic discs is related to the susceptibility of the organisms toward enoxacin and its metal complexes (Tables 3, 4, and 5). Standard deviation values amongst replicate responses were <0.6 indicating precision of the disc diffusion method.

It was observed that Nickel(II) complex exhibited increased antimicrobial effect than enoxacin against all the test strains except *Staphylococcus aureus*. Manganese(II) complex showed improved activity against *Staphylococcus aureus * and *Bacillus subtilis * while Copper(II) complex proved to be more active against *Citrobacter * and *Staphylococcus aureus*. Nearly all metal complexes showed increased activity as compared to enoxacin against *Clostridium hofmani * except Iron(III) complex, which was found to possess reduced activity against all the test strains as compared to enoxacin itself. Activity of Manganese(II) and Copper(II) complexes was depressed against *Escherichia coli*, *Pseudomonas aeruginosa * and * Salmonella typhi * while against *Streptococcus pneumoniae*, *Klebsiella pneumoniae, Shigella flexneri, * and *Proteus mirabilis * no appreciable change in activity was observed probably due to the intracellular biological conversion of the complexes [25].

 The increased activity of metal chelates can be explained on the basis of the overtone concept and chelation theory. According to the overtone concept of cell permeability, the lipid membrane that surrounds the cell favors the passage of only lipid-soluble materials in which liposolubility is an important factor that controls the antimicrobial activity. On chelation the polarity of the metal ion will be reduced to a greater extent due to overlap of ligand orbital and partial sharing of the positive charge of the metal ion with donor groups. Further it increases the delocalization of *π*-electrons over the whole chelate ring and enhances the lipophilicity of complexes [26]. It is likely that the increased liposolubility of the ligand upon metal complexation may contribute to its facile transport into the bacterial cell which blocks the metal binding sites in enzymes of microorganisms. These complexes also disturb the respiration process of the cell and thus block the synthesis of proteins, which restricts further growth of the organism [24].

The antimicrobial activity of the metal salts was also investigated. It was found that the metal salts did not exhibit antimicrobial activity at the concentration range used to assay the activity of the complexes in our work [22, 27]. 

### 3.2. Effect on Phagocytes Oxidative Burst

In order to test the immunomodulatory effect of the drug and its metal complexes, we investigated their effect on the oxidative burst activity of whole blood phagocytes. Phagocytic cells on activation induce release of reactive oxygen free radicals (oxidative burst) which is then quantified by a luminol-enhanced chemiluminescence assay. Results indicate that the zymosan- induced oxidative burst in whole blood phagocytes was inhibited (up to 50%) by Manganese(II) complex at a concentration of 15.3 ± 0.7 (*μ*g/mL) while Copper(II) complex produced 50% inhibition at a concentration of 18.7 ± 2.1 (*μ*g/mL). Both enoxacin and its Iron(III) and Nickel(II) complex produced 50% inhibition at a concentration above 50 (*μ*g/mL). Hence it is inferred that only Manganese(II) and Copper(II) complexes showed moderate inhibitory activity with IC_50_ ranging between 15–19 *μ*g/mL while the drug and other complexes have negligible effect on the oxidative burst response. The molecular mechanism causing the immunomodulatory effects of fluoroquinolones and their complexes are still under investigation [28].

## 4. Conclusion

Many drugs possess modified toxicological and pharmacological properties when in the form of metal complexes [29]. Present work deals with the synthesis of enoxacin metal complexes and the evaluation of the synergistic or antagonistic behavior of these complexes in comparison to the parent, through the difference in their biological activities. The complexes showed a diverse antimicrobial activity as compared to enoxacin which is attributed to the formation of metal drug chelates. Although complexation with metals is known to boost biological activities of the quinolones; yet in the form of Iron(III) complex, activity of enoxacin was immensely decreased which may produce a detrimental effect in its therapeutic efficacy. Nevertheless, complexation with Nickel(II) resulted in improved antimicrobial profile of the drug while Manganese(II) and Copper(II) metal complexes modulated oxidative burst response of phagocytes and unlike enoxacin could have potential to be anti-inflammatory, as they suppressed the production of reactive oxygen species.

## Figures and Tables

**Table 1:** Physicochemical Parameters of Enoxacin and its metal complexes.

S.No	Complexes	Color	Melting point °C	Mole Ratio	% yield
1	Enoxacin	off white	225	—	
2	Mn^II^ complex	off white	265d	1 : 2	78
3	Fe^III^ complex	dark brown	275d	1 : 1	70
4	Ni^II^ complex	leaf green	246	1 : 2	76
5	Cu^II^ complex	light blue	255	1 : 2	72

**Table 2:** CHN Microanalysis of Enoxacin and its metal Complexes.

S.No	Compound name		C	H	N	Chloride	Metal
1	Enoxacin	Found	56.42	5.85	16.99	—	—
		Calculated	56.24	5.35	17.49		
2	[Mn(eno)_2_(H_2_O)_2_]·3H_2_O	Found	45.40	6.12	14.04	—	7.05
		Calculated	45.90	5.92	14.34	—	6.99
3	[Fe(eno)(H_2_O)_2_]·Cl·4H_2_O	Found	33.67	5.27	10.47	13.2	10.43
		Calculated	33.73	5.20	10.58	13.5	10.24
4	[Ni(eno)_2_(H_2_O)_2_]·3H_2_O	Found	45.53	5.74	14.01	—	7.77
		Calculated	45.65	5.62	14.19	—	7.43
5	[Cu(eno)_2_(H_2_O)_2_]·3H_2_O	Found	45.39	5.56	14.12	—	8.02
		Calculated	45.37	5.58	14.11	—	8.00

**Table 3:** Zone of inhibition (mm) of enoxacin and its metal complexes.

Sample	*Clostridium hofmani*			*Staphylococcus aureus*			*Bacillus subtilis*			*Shigella flexneri*		
	Conc. (mg/disc)			Conc. (mg/disc)			Conc. (mg/disc)			Conc. (mg/disc)		
	5	10	20	5	10	20	5	10	20	5	10	20
Enoxacin	8	14	16	8	10	14	8	10	12	8	11	13
Enox +Mn	10	16	20	15	20	21	12	15	16	8	9	13
Enox + Fe	8	10	10	10	10	10	7	9	11	7	8	10
Enox + Ni	14	18	24	7	10	10	10	12	16	10	11	15
Enox + Cu	11	13	17	14	15	20	10	11	11	8	11	12

**Table 4:** Zone of inhibition (mm) of enoxacin and its metal complexes.

Sample	*Pseudomonas areuginosa*			*Salmonella typhi*			*Streptococcus pneumonia*			*Citrobacter*		
	Conc. (mg/disc)			Conc. (mg/disc)			Conc. (mg/disc)			Conc. (mg/disc)		
	5	10	20	5	10	20	5	10	20	5	10	20
Enoxacin	12	16	18	12	17	21	9	12	14	10	12	14
Enox +Mn	9	12	15	10	15	18	9	12	14	10	10	11
Enox + Fe	8	8	10	9	10	12	8	10	12	10	12	12
Enox + Ni	13	18	20	11	17	22	10	12	22	14	15	18
Enox + Cu	10	11	12	8	12	14	11	13	13	14	16	18

**Table 5:** Zone of inhibition (mm) of enoxacin and its metal complexes.

Sample	*Escherichia coli*			*Klebsiella pneumonia*			*Proteus mirabilis*		
	Conc. (mg/disc)			Conc. (mg/disc)			Conc. (mg/disc)		
	5	10	20	5	10	20	5	10	20
Enoxacin	12	15	19	9	12	13	10	13	14
Enox +Mn	12	13	14	11	11	12	10	11	13
Enox + Fe	10	11	12	9	11	11	11	13	12
Enox + Ni	14	16	25	11	14	18	10	12	20
Enox + Cu	10	12	12	10	11	13	11	13	13
